# Repeated courses of low‐dose 2 × 2 Gy radiation therapy in patients with indolent B‐cell non‐Hodgkin lymphomas

**DOI:** 10.1002/cam4.2796

**Published:** 2020-04-06

**Authors:** Khalil Saleh, Jean‐Marie Michot, Antoine Schernberg, Julien Lazarovici, Claude Chahine, Alina Danu, Nadine Khalife‐Saleh, Julien Rossignol, David Ghez, Valentine Martin, Renaud Mazeron, Christophe Fermé, Angela Boros, Vincent Ribrag, Theodore Girinsky

**Affiliations:** ^1^ Department of Hematology Gustave Roussy Université Paris‐Saclay Villejuif France; ^2^ Drug Development Department Gustave Roussy Université Paris‐Saclay Villejuif France; ^3^ Department of Radiation Oncology Gustave Roussy Université Paris‐Saclay Villejuif France

**Keywords:** follicular lymphoma, indolent B‐cell non‐Hodgkin lymphoma, low‐dose radiotherapy, marginal zone lymphoma, primary cutaneous follicle center lymphoma

## Abstract

**Purpose:**

In patients with indolent B‐cell non‐Hodgkin's lymphoma (B‐NHL), one course of low‐dose radiotherapy (LD‐RT) 2 × 2 Gy is emerging as new option of therapy in palliative setting. Efficacy of LD‐RT when repeated remains to be determinate. This study aims to assess the efficacy of repeated LD‐RT given in patients with indolent B‐NHL.

**Materials and Methods:**

All consecutive adult patients who received two or more courses of LD‐RT 2 × 2 Gy for indolent B‐NHL at Gustave Roussy institution, during the period 1990‐2015 were retrospectively investigated.

**Results:**

Thirty‐three patients received two or more courses of LD‐RT for indolent B‐NHL during the study period. The median age was 57 (range 37‐80) years, histological types were distributed among follicular lymphoma (n = 24 pts; 73%), marginal‐zone lymphoma (n = 6 pts; 18%), and primary cutaneous follicle center lymphoma (n = 3 pts; 9%). The median number of low‐dose radiation therapy courses given per patients was 2 (range 2‐6). The overall response rates following the first and the second course of LD‐RT were 96% and 88%, respectively (*P* = .31). The 1‐ and 2‐years local control rates following the first courses of LD‐RT were 94% (CI 95: 86‐100) and 94% (CI 95: 86‐98); and were 91% (CI 95: 82‐100) and 88% (CI 95: 77‐100) following the second course of LD‐RT (*P* = .39).

**Conclusion:**

The repeated courses of LD‐RT offered similar efficacy compare with the first course in patients with indolent B‐NHL. LD‐RT repeated is a simple, easy to give, and non‐toxic asset that could be investigated as treatment option in patients with indolent B‐NHL.

## Graphical abstract

Low dose radiotherapy is a valuable and non‐toxic treatment option in the form of indolent lymphoma. The effectiveness of low‐dose radiotherapy is similarly maintained when repeated once or even twice (RT1, RT2 and RT3).

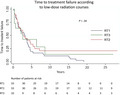

## 1 INTRODUCTION

B‐cell indolent non‐Hodgkin lymphomas (iNHL), especially follicular lymphoma (FL) and marginal zone lymphoma are highly radiosensitive disease.^1^, ^2^ For patients with localized lymphoma, involved field radiotherapy alone at 24 Gy was recognized as the treatment of choice in a curative intent.^3^ The long‐term outcome of radiotherapy alone in localized indolent lymphoma demonstrated relapse‐free survival in the range of 50% at 10 years,^4^ thus demonstrating that half of the patients can be considered cured with this treatment.^5^, ^6^

In a more conservative and palliative approach, lower doses of radiotherapy have been proposed in 2001, with a local 2 × 2 Gy over 3 days.^7^, ^8^, ^9^ The discovery that small doses of radiotherapy could eradicate low‐grade lymphoma was purely due to serendipity. One patient refused additional palliative course of radiotherapy after receiving 4 Gy, and at follow‐up he was found to be in complete response.^10^ This low‐dose radiotherapy (LD‐RT) was shown to be highly effective with a local control (LC) rate ranging from 82% to 94%, while maintaining a good quality of life with very few toxicities.^8^, ^9^, ^11^, ^12^, ^13^, ^14^, ^15^ This experience led to a non‐inferiority randomized trial, which finally showed that 4 Gy was inferior to 24 Gy in a curative intent.^16^ Although the dose of 24 Gy yielded better results, the results with 4 Gy were quite impressive with almost 50% of patients achieving a complete response and 32% having tumor regression.^16^ Thus, 24 Gy remains the standard dose for curative treatment of localized iNHL and in a symptomatic intent or in progressive setting, LD‐RT given at 2 × 2 Gy remains an excellent option of treatment.^3^ The LD‐RT was then used after failure of curative treatment or eventually for symptomatic intent in chemotherapy‐naïve patient.^1^, ^10^, ^11^, ^17^, ^18^, ^19^ Several studies have subsequently shown that some lymphoma subtypes were exquisitely sensitive to one course of LD‐RT such as FL,^10^ pulmonary mucosa‐associated lymphoid tissue lymphoma,^20^ primary cutaneous follicle center lymphoma (CFCL),^21^ and orbital marginal zone lymphoma.^22^

The LD‐RT regimen was investigated in many studies as a single course; however, little is known about the efficacy of LD‐RT when it is repeated two or more times. This study aims to assess the efficacy of repeated LD‐RT 2 × 2 Gy in patients with iNHL.

## 2 MATERIALS AND METHODS

### 2.1 Study design

All consecutive adult patients with iNHL who received two or more courses of LD‐RT between January 1990 and December 2015 at Gustave Roussy institution were included in this retrospective study. A minimum interval of 3 months was required between two courses of LD‐RT to consider that radiotherapy was of two distinct courses. The second course of LD‐RT could be delivered on the same anatomical site or at a different site. Patients who received any other concomitant systemic anti‐lymphoma treatment (in the 28 days before or after the LD‐RT) were not retained in the study. During the medical history and the follow‐up, all treatments given for lymphoma including immunotherapy, chemotherapy, targeted therapy, or radiotherapy at any doses were collected. Patients could have received anti‐lymphoma treatment before the first RT‐LD course, or between the different courses of LD‐RT. The selected histological types were indolent non‐Hodgkin B‐cell lymphoma of types FL, marginal‐zone lymphoma (MZL) or primary CFCL. All patients had to have a measurable disease staged with contrast‐enhanced computed tomography (CT), and were staged according to the Ann Arbor classification of Cheson 1999 criteria,^23^ or clinically measurable by photography with measurement for CFCL. Clinical and biological data, treatment characteristics, and outcome were collected until last follow‐up or death. This study was approved by the local institutional ethics committee board and all patients have given their consent for the data collection.

### 2.2 Radiation techniques and response assessment

The radiation techniques included simulation studies and planned immobilization devices were used according to treatment sites. According to local institutional guidelines, the planned dose was 4 Gy given in two fractions of 2 Gy over two or three consecutive days using cobalt unit or X‐rays beam of appropriate energy combine with electrons.^10^ The involved‐field radiation therapy technique was used. Margins varied with lesion size and body site and considered the dosimetry of the beam used. For primary CFCL and primary cutaneous marginal zone lymphoma, margin beyond the clinically obvious erythema or induration area Margin sizes ranged from 1.0 to 1.5 cm, in accordance with international recommendations.^24^

Local control was assessed 2 months after LD‐RT, by contrast‐enhanced CT in patients with imaging measurable lesions or by clinical examination and photography with measurement in superficial or visible lesion in patients with CFCL. According to the local institution guidelines after radiation therapy during the follow‐up period, patients were assessed every 3 months for 1 year by clinical examination and CT scan when clinically indicated, and then every 6 months.

### 2.3 Endpoints and statistical analysis

The main objective of the study was to assess the efficacy of a repeated LD‐RT as treatment of patients with indolent non‐Hodgkin lymphoma. The local responses rates with duration of local responses and systemic response outside the field of irradiation were investigated following the first course, second course and eventually subsequent courses of LD‐RT. End points were the LC rates and the treatment‐free survival (TFS). The overall survival (OS) was recorded. Patients were censored at the last visit of follow‐up or death and survival times were estimated using the Kaplan‐Meier method.^25^ Univariate analysis was performed to determine which factors were predictive of LC using log‐rank test. Differences in response rates were compared using the chi‐square test and the statistical analyses were performed using R software (version 3.3.2).

## 3 RESULTS

### 3.1 Characteristics of patients

Thirty‐three patients (pts), 14 women and 19 men, were included in the study. Median age was 57 years (range 37‐80). Histological types were distributed among FL (n = 24 pts; 73%), MZL (n = 6 pts; 18%), and CFCL (n = 3 pts; 9%) (Table 1). Patients received a median of two courses of LD‐RT (range 2‐6). The median time between the first LD‐RT course and the second one was 23.6 months (range 3‐144 months) and 16.6 months (range 4.9‐125.6 months) between the second course and the third one, when performed (*P* = .12). Seventeen of 35 patients (52%) received LD‐RT as a first‐line treatment for their iNHL, and the 16 remaining (48%) patients at relapse of lymphoma progression after systemic treatment.

**Table 1:** Characteristics of patients at initial diagnosis of indolent B‐cell non‐Hodgkin lymphoma, median number of low‐dose radiotherapy given and outcome

	Patients, n = 33 (% of total)
Age at diagnosis (y)	
Median	57
Range	37‐80
Sex	
Male	19 (58%)
Female	14 (42%)
Ann Arbor stage at diagnosis	
I	9 (27%)
II	8 (24%)
III	6 (18%)
IV	10 (31%)
Histological type	
Follicular lymphoma	24 (73%)
Marginal zone lymphoma	6 (18%)
Primary cutaneous follicle center lymphoma	3 (9%)
Anatomic site involved at diagnosis	
Abdomen	7 (21%)
Head and neck + abdomen	7 (21%)
Thoracic	5 (15%)
Head and neck	4 (12%)
Thoracic + head and neck + abdomen	3 (9%)
Cutaneous	3 (9%)
Thoracic + abdomen	2 (6%)
Head and neck + cutaneous	2 (6%)
Low‐dose radiotherapy courses	
Median number of courses per patient	2
Range	2‐6

Repetitive LD‐RT courses were given without any intercalated anti‐lymphoma systemic treatment between LD‐RT in 8 of 33 (24%) patients. A systemic anti‐lymphoma treatment between the separate courses of LD‐RT was given in 23 of 33 (70%) of patients. The two remaining (6%) patients received radiotherapy standard doses (24‐36 Gy) between the LD‐RT courses.

The anatomical sites targeted by the first course of LD‐RT were abdomen lymph nodes (n = 12 pts; 37%), head and neck lymph nodes (n = 8 pts; 24%), thoracic lymph nodes (n = 8 pts; 24%), cutaneous lymphomatosis lesions (n = 4 pts; 12%), and both thoracic and abdomen lymph nodes (n = 1 pt; 3%). The second LD‐RT course involved another anatomical site than first LD‐RT site in 30 of 33 (91%) patients and the same anatomical site in 3 of 33 (9%) of patients (Table 2).

**Table 2:** Characteristics of the first and the second courses of low‐dose radiotherapy

	n (%) of patients		*P* value^b^
	First low‐dose radiotherapy (n = 33 pts)	Second low‐dose radiotherapy (n = 33 pts)	
Number of irradiated site(s)			
1	20 (61)	24 (73)	.030
2	10 (30)	4 (12)	
3	1 (3)	4 (12)	
4	2 (6)	1 (3)	
Anatomic sites irradiated			
Abdomen	12 (37)	12 (37)	.970
Head and neck	8 (24)	7 (21)	
Thoracic	8 (24)	7 (21)	
Cutaneous	4 (12)	6 (18)	
Thoracic and abdomen	1 (3)	1 (3)	
Prior anti‐lymphoma treatment^a^			
Yes	16 (48)	18 (55)	.806
No	17 (52)	15 (45)	
Prior systemic immuno‐chemotherapy			
Yes	13 (39)	14 (42)	1.000
No	20 (61)	19 (58)	
Prior radiation therapy curative intension dose (24 Gy)			
Yes	7 (21)	9 (27)	.775
No	26 (79)	24 (73)	
Re‐irradiation by low‐dose radiotherapy in previous irradiated sites			
Yes	4 (12)	3 (9)	1.000
No	29 (88)	30 (91)	

a Other than low‐dose radiation therapy.

b Chi‐square test.

No acute or late toxicity were reported related to the LD‐RT courses. One patient had experienced diffuse large B cell lymphoma transformation during the follow‐up. With a median follow‐up of 12.0 years (range: 2.3‐24.5 years), the median OS was not reached (Figure S3 in Supporting Information). The estimated 5‐ and 10‐year OS were 100% and 88% (95% CI: 80%‐97%), respectively. Four patients had died during the follow‐up period, from lymphoma progression (n = 2 pts), heart coronary disease (n = 1 pt), and other malignancies hepatocellular carcinoma, (n = 1 pt). No death was related to radiotherapy toxicity.

### 3.2 Anti‐tumoral response following LD‐RT

The local responses and TFS following LD‐RT 2 × 2 Gy are shown in Table 3. Regarding all pooled LD‐RT courses, the estimated 1‐, 2‐ and 5‐year LC rates were 93% (95% CI: 88‐99), 92% (95% CI: 86‐98), and 87% (95% CI: 79‐95), respectively, and the median time to local recurrence was not reached.

**Table 3:** Best local response rates and treatment‐free survival following low‐dose radiotherapy 2 × 2 Gy

	All LD‐RT courses (76 courses)	1st LD‐RT course (33 courses)	2nd LD‐RT course (33 courses)	3rd LD‐RT course (10 courses)	*P* value^a^
Local response n (%) pts					
ORR	71 (93)	32 (96)	29 (88)	10 (100)	
CR	61 (80)	28 (84)	24 (73)	9 (90)	
PR	10 (13)	4 (12)	5 (15)	1 (10)	.31
SD	4 (6)	0 (0)	4 (12)	0 (0)	
PD	1 (1)	1 (3)	0 (0)	0 (0)	
Treatment free‐survival					
1‐y % (95% CI)	60 (50‐73)	61 (46‐80)	63 (49‐82)	50 (27‐93)	
2‐y (95% CI)	38 (28‐51)	36 (23‐57)	43 (29‐65)	27 (9‐81)	
3‐y (95% CI)	23 (15‐36)	18 (9‐38)	29 (16‐53)	27 (9‐81)	.34
Local‐control rate					
1‐y	93% (88‐99)	94% (86‐100)	91% (82‐100)	100% (NA)	
2‐y	92% (86‐98)	94% (86‐98)	88% (77‐100)	100% (NA)	
3‐y	87% (79‐95)	84% (73‐98)	88% (77‐100)	100% (NA)	.39

Abbreviations: CR, complete remission; LD‐RT, low‐dose radiation therapy 2 × 2 Gy; PD, progressive disease; PR, partial response; SD, stable disease.

a Chi‐square test.

**Figure 1:**
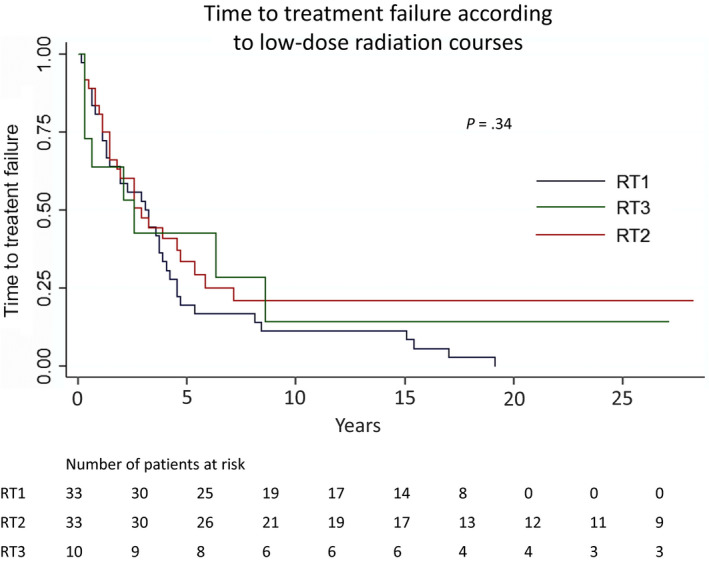
Time to treatment failure following the first, second, and third low‐dose radiation courses in patients with indolent non‐Hodgkin B‐cell lymphoma. RT1: first low‐dose radiotherapy 2 × 2 Gy course; RT2: second low‐dose radiotherapy 2 × 2 Gy course; RT3: third low‐dose radiotherapy 2 × 2 Gy course

Regarding all pooled LD‐RT courses, the overall response rate (ORR) was 93%, including 80% of complete responses and 13% of partial responses (Table 3). Similar responses rates were observed after the first and second course of LD‐RT (*P* = .31). The overall estimated 1‐ and 2‐year TFS were 60% (95% CI: 50‐73) and 38% (95% CI: 28‐51), respectively, and TFS were not statistically different comparing across first, second, and third LD‐RT courses (*P* = .34) (Figure 1). The median time to out‐field progression was 1.4 years (95% CI: 1.1‐2.0). Regarding the first, second, and third LD‐RT courses, the median time to out‐field progression was 1.6 years (95% CI: 1.0‐2.2), 1.5 years (95% CI: 1.1‐3.5), and 0.7 years (95% CI: 17‐NA), respectively, and were not significantly different (*P* = .34).

### 3.3 Outcome after the second course of LD‐RT

After the second course of LD‐RT, 16 of 33 patients (49%) received systemic treatment (immunotherapy, chemotherapy, or targeted therapies) or radiotherapy standard dose (24‐36 Gy), 11 of the 33 patients (33%) have not received a subsequent anti‐lymphoma treatment and were managed with a watch‐and‐wait approach, and 6 of the 33 patients (18%) received a third course of LD‐RT. The LD‐RT remains the last anti‐lymphoma treatment modality received at last follow‐up for 17 of 33 patients (52%) patients treated for FL (n = 10 pts), MZL (n = 4 pts), and CFCL (n = 3 pts).

### 3.4 Patients treated with exclusively repeated LD‐RT

The LD‐RT separate courses were given without any other intercalated anti‐lymphoma systemic treatment in 8 of 33 (24%) patients. These 8 patients were histologically distributed among FL (n= 5 pts) and MZL (n= 3 pts) lymphomas.

### 3.5 Predictive factors of response to LD‐RT

**Figure 2:**
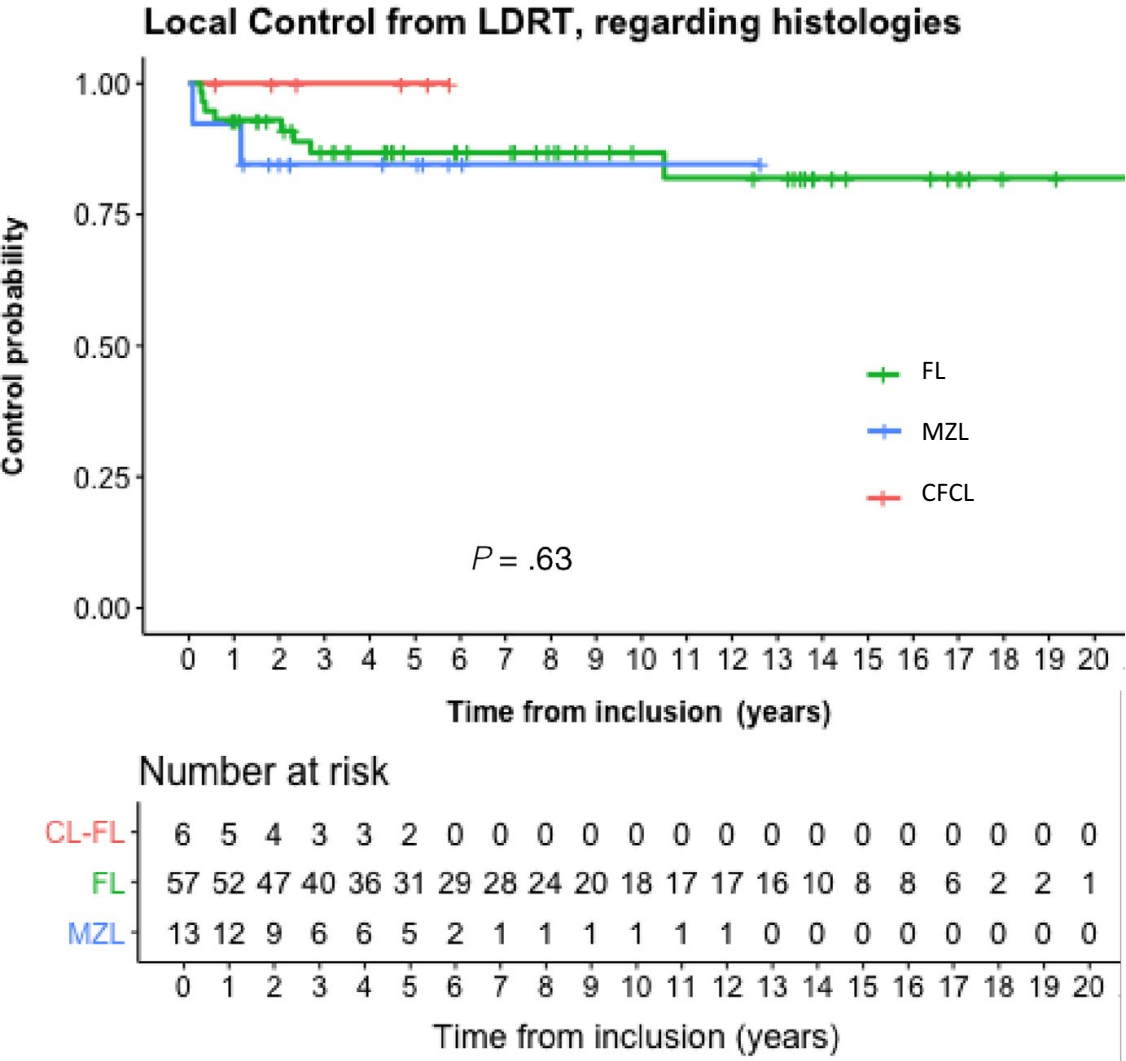
Local control following all courses (n = 76) of low‐dose radiotherapy given in all patients regarding histology types of B‐cell non‐Hodgkin lymphoma. CFCL, cutaneous follicle center lymphoma; FL, follicular lymphoma; MZL, mantle‐zone lymphoma

The LC rates after LD‐RT were similar among the different histologic type (Figure 2). None of the other factors studied (age, sex, Ann Arbor stage, number of prior regimens or time since diagnosis, anatomical sites of irradiation) were found to be related to the local response or TTF to LD‐RT including first or second courses of 2 × 2 Gy. Patients who underwent prior standard 24‐36 Gy radiotherapy had tendency of a worse LC rates after LD‐RT: the ORR was 33% (3/9 patients) in patients with previous standard radiotherapy vs 88% (21/24 patients) in patients without previous standard radiotherapy (*P* = .07) (data not shown).

## 4 DISCUSSION

The present series is the first to report the outcome and efficacy of repeated courses of low‐dose radiation therapies given in adult patients with indolent non‐Hodgkin B‐cell lymphoma. We found that the second and third low‐dose re‐irradiations have similar LC rates and similar duration of responses compare with the first LD‐RT course. With a median follow‐up of 12 years, we do not report any acute or late toxicity related to repeated low‐dose radiation therapy.

Many lymphoma types are notoriously sensitive to radiotherapy, especially the indolent types 1, 2, 26 marginal zone lymphoma or FL. When non‐Hodgkin lymphoma remains localized, as it is usually observed in two‐third of patients with marginal zone lymphoma and almost one‐third of patients with FL, radiotherapy is generally given within a potentially curative intent, even with low‐dose and small volumes are designed.^26^ In a curative intent, the recent randomized FORT study demonstrated that 24 Gy was superior to 4 Gy in the context of first line therapy for patients with a localized FL.^16^ However, 4 Gy radiotherapy was associated in this clinical trial with high responses rates and around one‐third of patients do not need further systemic therapy, indicating that this low‐dose therapy is an effective regimen, useful as easy to start and associated with virtually no toxicity. Thus, 24 Gy remains the standard dose in a curative treatment intent of localized disease, until a lower dose—that is between 4 and 24 Gy—need to be explored properly in further clinical trials.^3^

We have in our institution a very similar approach to treat indolent orbital lymphoma with a low dose 2 × 2 Gy. Almost all patients treated by this way for lymphoma of the orbital marginal zone have obtained a complete response. To date, no patient has required a second radiotherapy treatment (personal experience, data not shown).^1^ Johansson and Girinsky both reported an ORR of 81% and 82%, respectively,^9^, ^10^ the slight lower responses rates in these two studies may be explained by a higher proportion of patients with chronic lymphoid leukemia/small lymphocytic lymphoma, that is expected to be less sensitive to radiation.^9^, ^18^, ^27^ Interestingly, low‐dose radiation therapy was reported by Donaldson et al as a very effective and was advanced as an appropriate therapy for patients with a local indolent orbital or ocular adnexa lymphoma non‐Hodgkin lymphoma.^27^ After a single course of low‐dose radiation therapy, the authors found an ORR of 96%, and one of the 20 patients relapse and was successfully re‐irradiated at same low‐dose radiation.^22^ We have in our institution a very similar approach to treat orbital indolent lymphoma by low‐dose 2 × 2 Gy, and virtually all the patients treated for orbital marginal zone lymphoma achieved a complete response after a single course of low‐dose radiation and up‐to‐date none patient required a second course of radiation therapy (personal experience, data not shown).

We demonstrated in the present study that LD‐RT remains efficient as a second course after, even in patients that previously underwent LD‐RT to the same anatomical site. The similar LC rates regarding the first, the second, and third LD‐RT courses suggest that when 2 × 2 Gy is effective, it can be repeated several times with a renewed efficacy. Of note, the LC rate was found with a tendency of lower success in patients who have previously received a standard dose of 24‐36 Gy. The estimated 1‐ and 2‐year TFS were similar after first and second course of LD‐RT (*P* = .34) while we found a median time to out‐field progression of 1.4 year, which was consistent with Haas et al findings of 1.2 year.^1^ The median time to out‐field progression was also similar after first and second course of LD‐RT in our study. With the limitations of our retrospective study, these data suggest that repetitive LD‐RTs would not be associated with increased risk of local or systemic progression.

Finally, we found that 24% (8 of 33) patients in our series received only LD‐RT given as repeated in the course of their disease, with any other standard dose of radiotherapy neither systemic therapy, with a median follow‐up of 12 years. This result indicates that nearly a quarter of patients with indolent non‐Hodgkin lymphoma in our series could be spared from an exposure to systemic treatment such as chemotherapy or full‐dose radiation therapy.

Girinsky et al previously found predictive markers of responses to LD‐RT were patient age (<65 years), a not‐bulky tumor size (<5 cm) and a number of previous lines of chemotherapy 0‐1.^10^ Luthy et al reported the site of irradiation was associated with response, with more complete responses found in head and neck compare with pelvic or inguinal‐femoral localizations.^14^ Of note, in our study we were not able to accurately analyze bulky tumor size as risk factor due to missing data. Furthermore, in our study, we did not find any significant predictor associated with the antitumor response. This should be due to the lack of statistical power due to the small number of patients in our study.

We believe that LD‐RT should be an attractive therapy when conbined with immune checkpoint inhibitors. Indeed, this combination could potentially generate a synergistic or abscopal anti‐tumor effect.^28^

We acknowledge the main limitations of the study were due to the retrospective report and the limited number of patients, as well as the single experience design of the study. The main bias in our study was due to its retrospective design with selection bias, as the chosen population received local treatment for indolent lymphoma. It remains possible that some patients with the same characteristics and who did not receive treatment (watch and wait strategy) could have had the same outcome.

We believe that repetition of LD‐RT in the context of patients that do need a curative therapy for indolent follicular, cutaneous B‐cell indolent lymphoma or marginal zone lymphoma is an option of therapy to consider. Repeated LD‐RT for patients requiring therapy for indolent follicular, cutaneous B ‐ cell lymphoma or marginal zone lymphoma is a therapeutic option that is practical to consider. Repeated LD‐RT therapy may delay the need for a systemic therapy in some patients with indolent non‐Hodgkin's B‐cell lymphoma. Prospective clinical trials are needed to determine which patient populations may benefit from these low‐dose radiation therapies.

## 5 CONCLUSION

A second course of LD‐RT offered similar efficacy compare with a first course, in patients with indolent B‐cell non‐Hodgkin's lymphoma. LD‐RT is a simple, nontoxic, rapid and easy‐to‐initiate treatment for patients with indolent B‐cell non‐Hodgkin's lymphoma, and deserves further investigation.

## CONFLICT OF INTEREST

Jean‐Marie Michot: Principal/sub‐Investigator of Clinical Trials for: Abbvie, Aduro, Agios, Amgen, Argen‐x, Astex, AstraZeneca, Aveo pharmaceuticals, Bayer, Beigene, Blueprint, BMS, Boeringer Ingelheim, Celgene, Chugai, Clovis, Daiichi Sankyo, Debiopharm, Eisai, Eos, Exelixis, Forma, Gamamabs, Genentech, Gortec, GSK, H3 biomedecine, Incyte, Innate Pharma, Janssen, Kura Oncology, Kyowa, Lilly, Loxo, Lysarc, Lytix Biopharma, Medimmune, Menarini, Merus, MSD, Nanobiotix, Nektar Therapeutics, Novartis, Octimet, Oncoethix, Oncopeptides AB, Orion, Pfizer, Pharmamar, Pierre Fabre, Roche, Sanofi, Servier, Sierra Oncology, Taiho, Takeda, Tesaro, Xencor. PERSONAL FEES (Monies paid to you for services rendered, generally honoraria, royalties or fees for consulting, lectures, speakers bureaus, expert testimony, employment, ad‐boards, etc): Roche, Celgene, Bristol‐Myers Squibb, AstraZeneca, Janssen. Vincent Ribrag: Principal/sub‐Investigator of Clinical Trials for: Abbvie, Aduro, Agios, Amgen, Argen‐x, Astex, AstraZeneca, Aveo pharmaceuticals, Bayer, Beigene, Blueprint, BMS, Boeringer Ingelheim, Celgene, Chugai, Clovis, Daiichi Sankyo, Debiopharm, Eisai, Eos, Exelixis, Forma, Gamamabs, Genentech, Gortec, GSK, H3 biomedecine, Incyte, Innate Pharma, Janssen, Kura Oncology, Kyowa, Lilly, Loxo, Lysarc, Lytix Biopharma, Medimmune, Menarini, Merus, MSD, Nanobiotix, Nektar Therapeutics, Novartis, Octimet, Oncoethix, Oncopeptides AB, Orion, Pfizer, Pharmamar, Pierre Fabre, Roche, Sanofi, Servier, Sierra Oncology, Taiho, Takeda, Tesaro, Xencor. PERSONAL FEES (Monies paid to you for services rendered, generally honoraria, royalties or fees for consulting, lectures, speakers bureaus, expert testimony, employment, ad‐boards, etc): Medimmune, Pfizer, Servier, AstraZeneca, Janssen Bristol‐Myers Squibb. Other authors: nothing to disclose.
