# Supplementary figures and images for: Repeated courses of low‐dose 2 × 2 Gy radiation therapy in patients with indolent B‐cell non‐Hodgkin lymphomas
Source: Cancer Med. 2020 Apr 6;9(11):3725–32. doi: 10.1002/cam4.2796 (PMC7286454; doi:10.1002/cam4.2796)

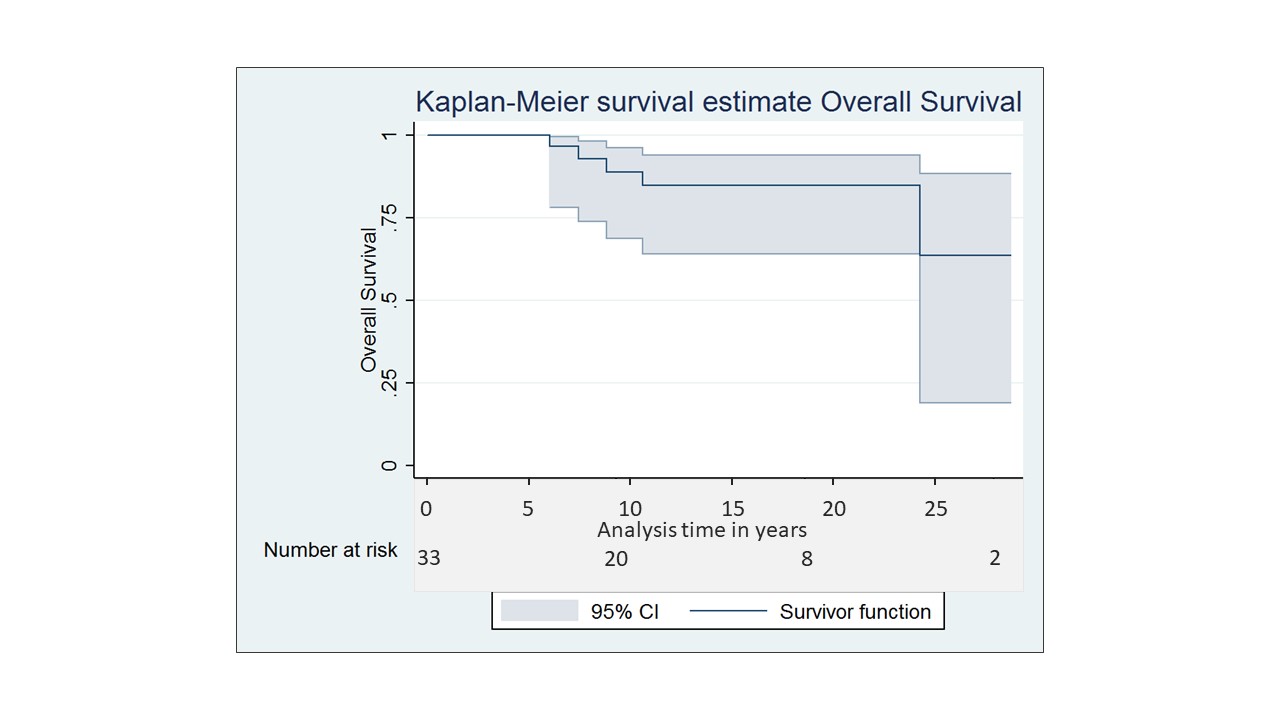

Supplement: Supplementary file 1 [file CAM4-9-3725-s001.jpg]
